# Investigation of the factor structure of GAD-7 in Moscow residents exposed to SARS-CoV2

**DOI:** 10.1192/j.eurpsy.2024.1052

**Published:** 2024-08-27

**Authors:** M. Zinchuk, G. Kustov, V. Nadezhda, A. Razmakhnin, D. Zhuravlev, R. Akzhigitov, A. Guekht

**Affiliations:** ^1^ Moscow Research and Clinical Centre for Neuropsychiatry; ^2^Pirogov Russian National Research Medical University, Moscow, Russian Federation

## Abstract

**Introduction:**

Rates of anxiety in the general population increased significantly during the COVID-19 pandemic. Several studies have shown that people exposed to SARS-CoV2 are at increased risk for both exacerbation and de novo development of anxiety disorders. Therefore, screening for anxiety disorders in this at-risk population is essential. In pre-pandemic studies, the 7-item Generalized Anxiety Disorder Questionnaire (GAD-7) was one of the most commonly used self-report instruments. Its validity has been demonstrated in several studies. However, there is no agreement among researchers about its underlying internal structure. Both one-factor and two-factor solutions have been reported. This discrepancy may be due to linguistic, cultural, and clinical differences between the populations studied. To our knowledge, no studies have been conducted to investigate the factor structure of the GAD-7 in the Russian-speaking community sample and the psychometric properties of this questionnaire in SARS-CoV2 exposed individuals.

**Objectives:**

The aim of the study was to determine the factorial structure and internal consistency of the Russian version of the GAD-7 in a large sample of Moscow residents exposed to SARS-CoV2.

**Methods:**

Fourteen thousand 725 (male - 11479 (78.0%), age - 18-79 years (M - 47.09, SD - 12.70) Moscow residents exposed to SARS-CoV2 completed an online survey including the GAD-7 and an ad hoc questionnaire focusing on socio-demographic characteristics. McDonald’s Omega was used to assess internal consistency. Exploratory structural equation modelling (ESEM) with weighted least squares means and variance adjusted estimator and geomin rotation was used to assess the factor structure of the Russian version of the GAD-7.

**Results:**

The McDonald’s Omega of the Russian version of the GAD-7 was 0.85, indicating a good internal consistency of the questionnaire. ESEM provided evidence for a one-factor solution that fits the data well (CFI - 0.996; TLI - 0.995; RMSEA (95% CI) - 0.041 (0.037 - 0.045)).

**Conclusions:**

In Russian people exposed to SARS-CoV2, the GAD-7 showed good internal consistency. Our results are consistent with those of previous studies that reported a single-factor solution for the questionnaire.

**Disclosure of Interest:**

None Declared

